# Heavy Metal Pollution in Settled Dust Associated with Different Urban Functional Areas in a Heavily Air-Polluted City in North China

**DOI:** 10.3390/ijerph13111119

**Published:** 2016-11-10

**Authors:** Dejun Wan, Zhangxiong Han, Jinsong Yang, Guanglin Yang, Xingqi Liu

**Affiliations:** 1 College of Resource Environment and Tourism, Capital Normal University, Beijing 100048, China; xqliu@niglas.ac.cn; 2 Provincial Key Laboratory of Mineral Exploration and Utilization, Xi’an 710054, China; han10260@163.com; 3 Institute of Hydrogeology and Environmental Geology, Chinese Academy of Geological Sciences, Shijiazhuang 050061, China; yjs607@163.com (J.Y.); yucb8688@163.com (G.Y.)

**Keywords:** heavy metal, atmospheric particle, enrichment factor, integrated pollution index, different functional area, spatial distribution, Shijiazhuang

## Abstract

Understanding variations of heavy metals in atmospheric particles between different functional areas is significant for pollution control and urban planning in cities. To reveal pollution and spatial distribution of heavy metals in atmospheric particles from different urban functional areas in Shijiazhuang in North China, 43 settled dust samples were collected over the main urban area and heavy metal concentrations were determined in their <63 μm fractions using an ICP-OES. The results suggest that Cr, Mn, Fe, Co, Ni, and V in the dust are not or slightly enriched and their concentrations vary slightly between different sites, implying their natural origins; whereas Cu, Zn, Cd, and Pb are often notably enriched and their concentrations vary significantly between different functional areas, indicating their anthropogenic sources. Integrated pollution indexes (IPIs) of the ten heavy metals are 2.7–13.6 (5.7 ± 2.2), suggesting high or very high pollution levels of most dust. Relatively lower IPIs occur mainly in the administration-education area, the commercial area, and other unclassified sites; while peaks occur mainly in the North Railway Station, the northeastern industrial area, and some sites near heavily trafficked areas, implying the significant influence of intensive industrial (including coal combustion) and traffic activities on atmospheric heavy metal accumulation. These results suggest a clear need of mitigating atmospheric heavy metal pollution via controlling emissions of toxic metals (especially Cd and Pb) from industrial and traffic sources in the city.

## 1. Introduction

Heavy metals adhered on atmospheric particles not only lead to health impacts for humans and other living beings via direct inhalation, ingestion and dermal contact absorption, but also impose a long-term burden on environmental quality in aquatic and terrestrial ecosystems [1,2,3]. Among all the heavy metals, lead (Pb), arsenic (As), mercury (Hg), cadmium (Cd), and chromium (Cr) are of the greatest concern for public health because of their high degree of toxicity [2,4,5]. Heavy metals in atmospheric particles originate from natural sources such as crustal minerals, volcanic eruptions, forest fire, etc., but most environmental pollution and human exposures are caused by anthropogenic activities such as mining, smelting, industrial production and use, vehicular traffic, fossil fuel combustion, domestic and agricultural use of metals and metal-containing compounds, and so on [2,3,4].

Owing to accelerated industrialization and urbanization development in recent decades, more and more anthropogenic metals have been emitted into the atmosphere with heavy metal pollution accidents reported frequently in China in recent years [2,4]. Generally, heavy metals are often more enriched in atmospheric particles from urban areas than those from rural areas less affected by anthropogenic activities [2]. In addition, urban areas are often densely populated. In 2016, 49.68% of people (~666 million) lived in urban areas in China. Hence, heavy metals in atmospheric particles from large cities have attracted great attention. In recent years, a lot of valuable studies have been conducted to investigate the pollution status, temporal and spatial variation, sources, causes, control measures, etc. of heavy metals in atmospheric particles in many large cities in China such as Beijing, Tianjin, Shanghai, Guangzhou, Xi’an, etc. [6,7,8,9,10]. However, most of these investigations were often limited to collect samples at only one or limited sites in a city due to the use of the traditional filter sampling method which needs heavy workload. Heavy metals in atmospheric particles from different urban functional areas often vary significantly owing to the different activities carried out in them, such as industry, commerce, culture and education [6,11,12,13,14]. It may therefore be difficult to accurately understand heavy metal pollution status in these cities due to the settlement of atmospheric particles and to reveal heavy metal variations between different urban functional areas.

To reveal heavy metal variations in atmospheric particles between different urban functional areas, in this study Shijiazhuang city was chosen to conduct this investigation as it is a heavily air-polluted area representative of the main industrialized urban areas in China [9]. Based on 43 settled dust samples collected from typical different urban functional areas in Shijiazhuang, concentrations of ten heavy metals were determined in their <63 μm fractions. This fraction is roughly similar to total suspended particulate matter (TSP) which has received worldwide attention due to its variously adverse effects on human and ecosystem health [15]. The major objectives of this study are: (1) to understand pollution levels of heavy metals in atmospheric particles in Shijiazhuang city; (2) to reveal variations of heavy metal pollution between different functional areas; and (3) to identify major sources of heavy metals in the city. Such a study is significant for heavy metal pollution control and urban planning in Shijiazhuang, for understanding spatial patterns of atmospheric heavy metals in other similar cities in China, and for source apportionment of heavy metals in atmospheric particles.

## 2. Materials and Methods

### 2.1. Study Area

**Figure 1:**
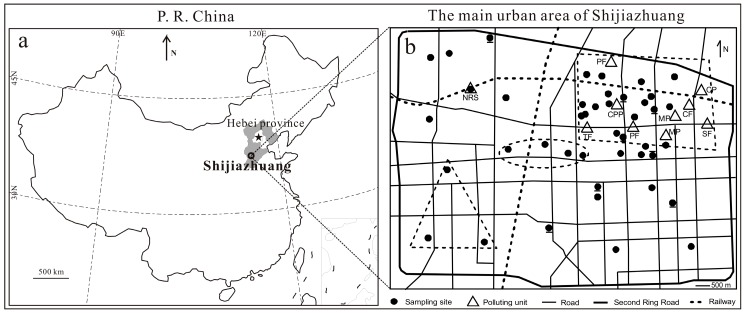
Location of Shijiazhuang in China (**a**) and sampling sites (solid dots) for 43 settled dust collected over the main urban area (**b**). Solid dots with short lines on the bottom or the top represent sampling sites near heavily trafficked areas. The dotted triangle, ellipse, and quadrangle in (**b**) represent the administration-education area, the commercial area, and the old industrial area, respectively. Triangles in (**b**) representative polluting units in the study area. NRS, PF, CPP, TF, MP, SF, CF, and CP in (**b**) represent the North Railway Station, pharmaceutical factory, coal-fired power generation plant, textile factory, machinery plant, steel and iron factory, coking factory, and chemical plant, respectively. However, it should be noted that, besides these polluting units shown in the figure, there are also dozens of other relatively small polluting units distributed in the study area.

The main urban area of Shijiazhuang city covers a flat area of around 120 km^2^. It is geographically situated at 114.4° E–114.6° E and 38.0° N–38.1° N on the western North China Plain (Figure 1a). Shijiazhuang is the capital of Hebei Province, and has a population of 10.16 million (2010), with about a third of the people living in the main urban area. It is a traditional industrial base in China, with priori developments of steel and iron, pharmacy, textile, metallurgy, mechanical manufacturing and processing, coal industry, and so on. The urban area is divided into different functional sub-areas such as industry, commerce, administration-education, etc.

The climate belongs to the warm temperate continental monsoon climate in the city, with a precipitation of about 500 mm/year, most (70%) of which occurs in the summer months between June and August. In recent years, haze events occur frequently and air pollution is getting worse [9]. According to the annual report issued by the Ministry of Environmental Protection in China [16,17], in 2013 the five top air-polluted cities in China were Xingtai > Shijiazhuang > Handan > Tangshan > Baoding; in 2014 they were Baoding > Xingtai > Shijiazhuang > Tangshan > Handan. Therefore, the serious pollution status of the atmospheric environment should arouse attention and effective measures should be taken for improving air quality in Shijiazhuang.

### 2.2. Sampling and Analysis

In this study, 43 settled dust samples were collected from different functional areas over the main urban area of Shijiazhuang in May 2013, i.e., sixteen dust samples were from the industrial area, eight near heavily trafficked areas, four from the commercial area, three from the administration-education area, one at the North Railway Station, and eleven from other unclassified sites (Figure 1b). Generally, relatively large residential quarters were chosen for dust sampling at most of these sites. At each site, buildings that were relatively far from point-source polluting sources were chosen for dust sampling. The dust samples were collected, using a small brush and a plastic dustpan [18,19,20,21], mainly from uncleaned outdoor surfaces of windowsills, flat roofs, pipelines, etc. on buildings which were >5 m (most >10 m) high above the ground and seldom disturbed by rain and human activities [15,20]. The dust samples were collected from several sub-sites at most of the sampling sites including the North Railway Station. Such sampling methods could improve the representativeness, to some extent, of heavy metals in the dust.

Concentrations of heavy metals (Cr, Mn, Fe, Co, Ni, Cu, Zn, Cd, Pb and V) and Al in the settled dust samples were measured using an Inductively Coupled Plasma Optical Emission Spectrometry (iCAP 6300, Thermo Fisher Scientific, Waltham, MA, USA). Before measurement, the dust samples were sieved with a ~63 μm (250 mesh) pore-size sieve, and only <63 μm fractions were used for heavy metal measurements. After being dried in an oven (101-2AB, Taisite Instrument, Tianjin, China) at 105 °C for 8–12 h, ~0.25 g dust was weighed and hot-digested with concentrated HNO_3_, HClO_4_, and HCl in Teflon vessels on a hot plate [22]. The detection limits of the method for Cr, Mn, Fe, Co, Ni, Cu, Zn, Cd, Pb, V, and Al are 1.7, 0.6, 19, 0.01, 0.5, 0.06, 1.5, 0.04, 0.3, 0.07, and 24 mg·kg^−1^, respectively. Blanks and duplicate measurements were measured to ensure the quality and accuracy of the sample pretreatment and measurement processes. The recovery rates for the eleven elements relative to the standard reference material (GSB 04-1767-2004) ranged between about 96% and 105%. Replicate analyses suggested that the precision of these analyses was approximately <5% relative standard deviation at a 95% confidence level.

### 2.3. Enrichment Factor (EF)

Enrichment factor (EF) is one of the most commonly used indexes to assess enrichment or pollution of heavy metals in environmental media and to evaluate their natural and anthropogenic sources [15,18,21]. The EF value of a metal in a dust sample can be calculated using Equation (1):

EF = (X*_i_*/X*_r_*)_dust_/(X*_i_*/X*_r_*)_natural·soil_ (1)

where X*_i_* is the concentration of metal *i* and X*_r_* is the concentration of the reference element (Al) in a dust sample or in the local natural soil. Metal concentrations in the local natural soil were determined based on 148 surface soil samples collected from sites that were seldom affected by human activities over Hebei Province [23]. Based on the calculated EFs, the enrichment of heavy metals in dust can be divided into the following five classes [15,18,21]: (1) EF ≤ 2, low enrichment; (2) 2 < EF ≤ 5, moderate enrichment; (3) 5 < EF ≤ 20, high enrichment; (4) 20 < EF ≤ 40, very high enrichment; and (5) EF > 40, extremely high enrichment.

### 2.4. Integrated Pollution Index

To assess the integrated pollution status of all the detected heavy metals in the dust, an integrated pollution index (IPI) was used in this study. The IPI of all the heavy metals in a dust sample can be calculated using Equation (2) [11,24,25,26]:

IPI = (PI_1_ + PI_2_ + … + PI*_i_* + … + PI*_n_*)/*n* (2)

where PI*_i_* represents the pollution index of the metal *i* and *n* represents the number of heavy metals studied in the dust. The PI*_i_* is the ratio of metal concentration (C*_i_*) relative to that in the local natural soil (B*_i_*) [23], which can be calculated using Equation (3):

PI*_i_* = C*_i_*/B*_i_* (3)

The PI of a metal can be classified as [11,24,25,26]: (1) PI < 1, a low pollution level; (2) 1 < PI < 3, a moderate pollution level; and (3) PI > 3, a high pollution level. The IPI of all the heavy metals in a dust can be classified as: (1) IPI ≤ 1, a low pollution level; (2) 1 < IPI ≤ 2, a moderate pollution level; (3) 2 < IPI ≤ 5, a high pollution level; and (4) IPI > 5, a very high pollution level.

## 3. Results and Discussion

### 3.1. Heavy Metal Concentrations in the Settled Dust

Statistical values of heavy metal concentrations in the <63 μm fractions of the 43 settled dust from the Shijiazhuang urban area are shown in Table 1. Additionally, concentrations of these metals in the local natural soil from Hebei Province [23], the Upper Continental Crust (UCC) [27], and dust from representative cities in China [12,15,28,29,30] are also given in the table. The average concentrations of Cr, Mn, Fe, Co, Ni, Cu, Zn, Cd, Pb, and V in the settled dust are 141.4 ± 41.2, 562.3 ± 102.2, 43,348 ± 10,692, 14.9 ± 3.3, 42.1 ± 13.7, 107.3 ± 47.9, 514.5 ± 216.6, 2.84 ± 1.55, 177.0 ± 99.9, and 65.9 ± 14.9 mg·kg^−1^, respectively. The concentrations of Mn, Fe, Co, Ni, and V in the settled dust are close to those in the natural materials (local soil [23] and UCC [27]), whereas that of Cu, Zn, Cd, and Pb are obviously higher compared to the natural materials. The average concentrations of Cu, Zn, Cd, and Pb in the settled dust are 4.9, 6.6, 30.2, and 8.2 times higher compared to those in the local natural soil [23], respectively. This implies that Mn, Fe, Co, Ni, and V in the dust are likely derived dominantly from natural sources, whereas Cu, Zn, Cd, and Pb seem to be influenced by others sources. As for Cr, although its average concentration in the settled dust is twice times higher than that in the local natural soil, it also is likely affected mainly by natural sources according to the EF and cluster analysis results in next part.

Compared to dust from other representative cities in China (Table 1), the average concentrations of Mn, Co, Ni, Cd, and V in the settled dust from Shijiazhuang are close to that from the other cities; that of Cr is close to that from Xi’an [29], Wuhan [12], and Chengdu [30], but higher than that from Beijing [15] and Changchun [28]; that of Cu is close to that from Beijing [15] , Wuhan [12], and Chengdu [30], but higher than that from Changchun [28] and Xi’an [29]; that of Zn is close to that from Changchun [28] and Xi’an [29], but lower than that from Beijing [15], Wuhan [12], and Chengdu [30]; that of Pb is close to that from Beijing [15] and Xi’an [29], lower than that from Wuhan [12], but higher than that from Changchun [28] and Chengdu [30]. This comparison suggests a moderate pollution level of heavy metals in the urban dust from Shijiazhuang among representative cities in China, in agreement with the developing economic status of Shijiazhuang.

### 3.2. Enrichment Factors of Heavy Metals

**Figure 2:**
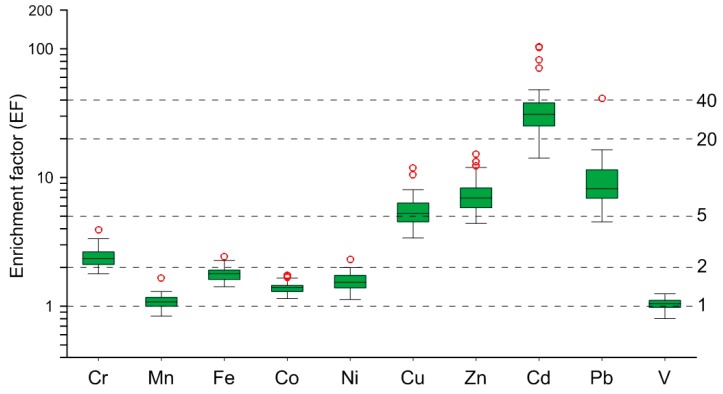
Box-plots for the enrichment factors (EF) of heavy metals in the 43 settled dust samples from the Shijiazhuang urban area relative to the local natural soil (Al was chosen as the reference element).

Taking the local (Hebei Province) natural soil [23] as background, EF values of heavy metals in the settled dust are calculated and shown in Figure 2. The average EF values of the ten metals are in the decreasing order (Table 1): 40 > Cd > 20 > Pb > Zn > Cu > 5 > Cr > 2 > Fe > Ni > Co > Mn > V = 1 (Figure 2). According to the classification of enrichment, Mn, Fe, Co, Ni, and V are lowly enriched, Cr is moderately enriched, while Cu, Zn, Pb, and Cd are highly to very highly enriched in the settled dust from the Shijiazhuang urban area.

**Figure 3:**
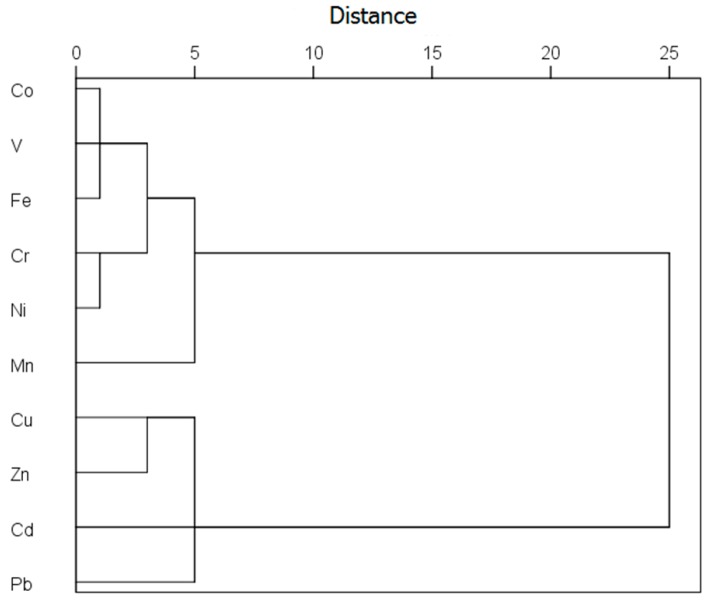
Hierarchical dendrogram for heavy metals in the 43 settled dust samples obtained by Ward’s hierarchical clustering method.

The above EF results, together with the cluster analysis results in Figure 3, suggest that these ten metals can be divided into two groups: (1) Cr, Mn, Fe, Co, Ni, and V and (2) Cu, Zn, Cd, and Pb. These results indicate that the six metals in the group 1 displaying low to moderate EF values are dominantly from natural sources and seldom affected by human activities while the four metals in group 2 with high EF values are mostly from anthropogenic sources [15,18,21].

### 3.3. Spatial Distribution and Sources of Heavy Metals in Dust from Different Functional Areas

**Figure 4:**
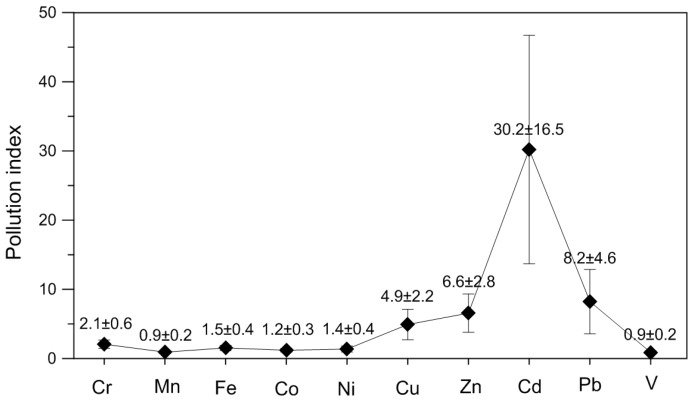
Pollution indexes (PIs) of heavy metals in the 43 settled dust samples from the Shijiazhuang urban area relative to the local natural soil.

Integrated pollution indexes (IPIs) of these ten heavy metals in the settled dust range from 2.7 to 13.6, with an average of 5.7 ± 2.2, suggesting high or very high pollution levels of these heavy metals in the dust samples. The pollution indexes (PIs) of Cr, Mn, Fe, Co, Ni, Cu, Zn, Cd, Pb, and V in the settled dust are 2.1 ± 0.6, 0.9 ± 0.2, 1.5 ± 0.4, 1.2 ± 0.3, 1.4 ± 0.4, 4.9 ± 2.2, 6.6 ± 2.8, 30.2 ± 16.5, 8.2 ± 4.6, and 0.9 ± 0.2, respectively (Figure 4). They are decreasing in the following order: Cd > Pb > Zn > Cu > Cr > Fe > Ni > Co > Mn = V. These results confirm a relatively lower pollution levels for the natural related elements (Cr, Mn, Fe, Co, Ni, and V) and notably higher pollution levels for the anthropogenic related elements (Cu, Zn, Cd, and Pb).

**Figure 5:**
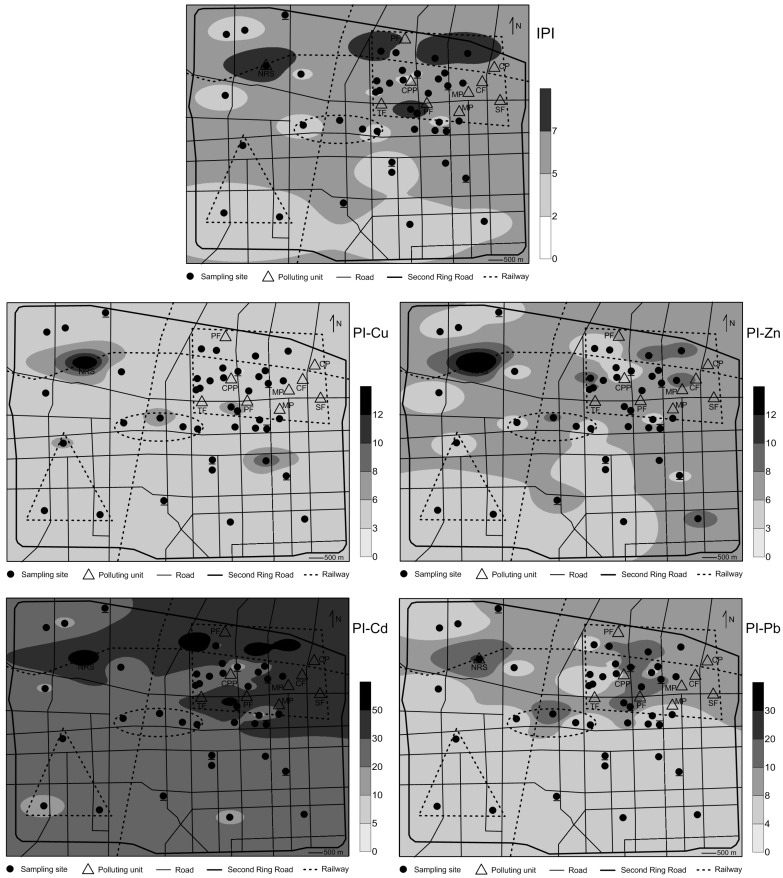
Spatial distributions for the integrated pollution indexes (IPIs) of the ten heavy metals and pollution indexes (PIs) of the four heavily polluted metals (Cu, Zn, Cd, and Pb) in the settled dust over the Shijiazhuang main urban area.

Overall, the IPIs of these ten heavy metals are between 5 and 7 at most sites of the urban area, suggesting a very high pollution level of these heavy metals in most dust, especially in dust from the industrial area and the North Railway Station (Figure 5). This implies that the modern urbanization and industrialization have caused a very high integrated pollution level of heavy metals in atmospheric particles from the Shijiazhuang urban area, even though some other important toxic elements (such as Hg and As) in the dust have not been considered in this study. According to the IARC (International Agency for Research on Cancer) regarding the toxicity of inhalable metals in atmospheric particulate matter, As, Cd, Cr, and Ni are classified Group 1: “Carcinogenic to humans” and Pb is classified Group 2A: “Probably carcinogenic to humans”. Considering these risks and the high concentrations of Cd and Pb, which are likely to be more concentrated in inhalable particles (PM_10_) relative to coarser ones [31], the serious pollution status of atmospheric particles in the Shijiazhuang urban area is concerning, and effective control measures need to be taken.

Among the ten heavy metals, Cd, Pb, Cu, and Zn are the dominant (86%) contributors to the IPIs, and PIs of these four metals vary significantly over the urban area. In contrast, the PIs of the other six metals (Cr, Mn, Fe, Co, Ni, and V) are not only relatively lower, but also of little spatial variability. Therefore, in the following only the IPIs and PIs of Cu, Zn, Cd, and Pb in the dust are discussed for their spatial distributions.

From the spatial distribution of the IPIs (Figure 5), it can be seen that relatively higher (>7) IPIs mainly occur in dust from a site in the northwestern urban area and from some sites in the northeastern urban area (Figure 5). The dust in the northwest was collected at the North Railway Station. The IPI (13.6) of this dust is the highest in the entire urban area. Concentrations from the different functional areas in Table 2 show that all the metals, especially Cu and Zn, from the Railway Station are obviously higher than those from the other areas. The Railway Station is one of the most heavily trafficked areas in the city. Every day there are more than a hundred trains, as well as large numbers of motor-vehicles such as buses, taxies, etc., passing in and out. Since most brake-linings used in cars contain high quantities (5%–20%) of Cu [32], the significant enrichment level of Cu in the dust at the Railway Station may be mainly related to the abrasion of vehicle braking devices. The friction of overhead cables of trains also can emit Cu into the atmosphere [32,33]. Generally, the enrichment of Zn in atmospheric particles is often related to intensive traffic activities, because of the high content of Zn in tyres, brake-linings, etc. of cars and trains [34]. Besides Cu and Zn, traffic activities also can emit large amounts of Pb, Cd, etc. [34]. These facts suggest the higher concentrations of heavy metals in the dust probably result from intensive train and automobile activities surrounding the Railway Station. Similarly, based on investigating heavy metals in re-suspended dusts over an oilfield city (Dongying) in China, Kong et al. [20] also found high concentrations of most metals (V, Cr, Mn, Co, Ni, Cu, Zn, As, Cd, and Pb) near a railway station related mainly to the influence of local vehicle emissions and fossil fuel combustion. Considering people (workers, passengers, and residents) in and near the Railway Station are potentially exposed to metal-rich particles, the high pollution levels of atmospheric heavy metals caused by traffic activities should be controlled as soon as possible.

The relatively higher IPIs in the northeastern urban area are probably caused by intensive industrial activities. The northeastern urban area is the old industrial area in the city. In this area there are dozens of heavily polluting units, such as the coal-fired power generation plant, metallurgy and machinery plant, steel and iron factory, coking factory, pharmaceutical factory, chemical plant, and so on (Figure 1b). Although the local government have started to take measures, such as carrying out stricter pollutant emission standards and even shutting down or relocating some heavily polluting units, to control industrial pollution in recent years, there are still many industry-related activities in this area and the atmospheric particulate matter pollution is still serious.

The spatial distributions of PIs in Figure 5 show that the PIs of Zn, Cd, and Pb in dust from this area are obviously higher compared to that from most other sites. It is suggested that Zn is the most common element released by the iron and steel industry; machinery and electroplating often release large amounts of Cd; and coal combustion for thermal power generation, metallurgy, coking, etc. is related to the release of many toxic metals such as Pb, As, Cu, Zn, etc. [1,2,4,5]. This implies that the higher pollution levels of Zn, Cd, and Pb in the atmospheric dust are mainly related to the industrial activities (including coal combustion). This fact suggests that the intensive industrial activities in the northeastern urban area are one of the most important sources for heavy metals in the Shijiazhuang urban area and thus it should be given special attention in the future environment management.

With respect to the dust collected near heavily trafficked and commercial areas, the levels of heavy metals are higher than those in the southwestern administration-education area and other unclassified sites, but lower than those from the North Railway Station and the northeastern industrial area (Figure 5 and Table 2). Although leaded gasoline was phased out in China in 2000 [35], the average Pb concentration in dust collected near heavily trafficked areas is higher than those in the other functional areas except for the North Railway Station (Table 2). This is probably as a result of: (1) historical residues from leaded gasoline combustion [22] and (2) additive effects of traffic and industrial activities in the northeastern urban area. As for dusts from the commercial area, only one collected in the center of this area shows relatively higher metal concentrations, especially for Pb (Figure 5). This is likely due to that it was collected near the largest wholesale market in the city where the traffic is extremely heavy and the environment is dirty and disorderly.

The lowest metal pollution is found in the southwestern administration-education area and the other unclassified sites (Figure 5 and Table 2), which is likely due to: (1) relatively fewer polluting units in these areas and (2) these areas being located far away from the industrial area in the city. However, heavy metal pollution in the atmospheric dust in these areas cannot be neglected, because the IPIs (between 2 and 5) are still high. This may be partly due to relatively long-distance diffusion of industrial and traffic emissions because <63 μm atmospheric particles collected at a height of >5 m above the ground can be easily transported from areas several kilometers away in favorable meteorological conditions [36].

Hebei province, where Shijiazhuang city is located, is one of the most important heavy industry bases in China. In this province, percentage of the high energy-consuming industry such as iron and steel, cement, etc. is high, and the energy efficiency is relatively low. Source apportionment results for atmospheric particles suggest that the most important local sources for PM_2.5_ are coal combustion (28.5%) (predominantly used for industry), industrial emissions (25.2%), fugitive dust (22.5%), and traffic activities (15.0%) in Shijiazhuang during February 2013–April 2014 [37]. Investigations of trace elements in atmospheric particles found that the enrichment of heavy metals mainly results from coal combustion (e.g., As, Hg, Cu, Pb), industrial emissions (e.g., Fe, Ni, Mn, Cu, Zn, Co, Cr) and traffic activities (e.g., Pb, Cu, Zn, Cd, Cr) in the regions of Beijing, Tianjin, and Hebei [3,7,8,38], similar to our study. These facts imply that the effective way to improve outdoor air quality and to mitigate atmospheric heavy metal pollution in the area is: (1) changing the energy-consuming industry, (2) reducing coal consumption and increasing energy efficiency, and (3) improving oil quality and controlling motor-vehicle movements.

## 4. Conclusions

Taking Shijiazhuang city, a heavily air-polluted city in North China, as an example, heavy metals in atmospheric particles were investigated in different urban functional areas of the city, based on analyzing the <63 μm fractions of 43 settled dust samples collected over the urban area. EF and cluster analysis suggest that these ten metals can be divided into two groups. The first group of Cr, Mn, Fe, Co, Ni, and V show similar (except for Cr) concentrations to that of the natural materials (local soil and UCC) and have lower EFs (1–2.5), implying predominantly natural sources. In contrast, the second group of Cu, Zn, Cd, and Pb is not only of higher concentrations relative to the natural materials but also of higher EFs (5–40), implying mostly from anthropogenic sources. Integrated pollution indexes (IPIs) of these ten heavy metals are between 2.7–13.6 and average 5.7 ± 2.2, suggesting high or very high pollution levels of these metals in most dust. The serious pollution status of heavy metals in atmospheric particles in the entire Shijiazhuang urban area should be controlled without delay. The dominant contributors for IPIs are Cd (52%), Pb (14%), Zn (11%), and Cu (8%). Comparisons of IPIs of heavy metals in dust between different functional areas suggest the highest pollution occurring at the North Railway Station and in the northeastern industrial area, a medium level near the heavily trafficked areas and in the commercial area, and the lowest in the southwestern administration-education area and other unclassified sites. The result indicates that industrial (including coal combustion) and traffic activities are the most important sources for heavy metals in atmospheric particles in the urban area. The concentrations observed in Shijiazhuang are comparable to those reported from other representative cities in China (Beijing, Changchun, Xi’an, Wuhan, and Chengdu). Consequently, this study is significant not only for environmental management and urban planning in the city, but also for understanding atmospheric heavy metal pollution in other similar cities in China.

## Figures and Tables

**Table 1:** Statistical results (mg·kg^−1^) and enrichment factor (EF) values for heavy metal concentrations in the <63 μm fractions of the 43 settled dust samples from the Shijiazhuang main urban area. Besides, average concentrations of heavy metals in the local natural soil, the Upper Continental Crust (UCC), and dust from other typical cities in China are also listed.

Location	Type	Item	Cr	Mn	Fe	Co	Ni	Cu	Zn	Cd	Pb	V	Data Source
			mg·kg^−1^										
Shijiazhuang	Settled dust (<63 μm)	Min	79.7	370.9	25,983	8.1	22.2	48.5	267.2	0.86	71.2	33.9	This study
		Max	279.6	852.1	78,899	26.2	82.9	330.1	1485.8	8.28	571.3	114.9	
		Median	137.9	556.0	42,715	14.1	39.2	99.6	466.9	2.40	146.9	63.7	
		Average	141.4	562.3	43,348	14.9	42.1	107.3	514.5	2.84	177.0	65.9	
		Stdev	41.2	102.2	10,692	3.3	13.7	47.9	216.6	1.55	99.9	14.9	
		EF	2.4 ± 0.4	1.1 ± 0.1	1.8 ± 0.2	1.4 ± 0.1	1.6 ± 0.2	5.7 ± 1.7	7.6 ± 2.7	38.7 ± 19.7	9.5 ± 5.7	1.0 ± 0.1	
Hebei	Natural soil	Average	68.3	608	28,200	12.4	30.8	21.8	78.4	0.09	21.5	73.2	[23]
UCC	-	Average	35	600	35,000	10	20	25	71	0.098	20	60	[27]
Beijing	Settled dust (<63 μm)	Average	86.0	607.1	-	10.6	45.2	138.4	722.7	2.29	167.9	57.9	[15]
Changchun	Surface dust (<0.8 mm)	Average	96.0	692.0	-	-	-	68.4	465.4	-	93.6	-	[28]
Xi’an	School dust (<75 μm)	Average	149.2	558.3	-	43.4	34.6	70.8	461.5	-	180.9	67.6	[29]
Wuhan	Surface dust (<250 μm)	Average	130	-	-	14	33	138	835	-	281	-	[12]
Chengdu	Outdoor Dust (<63 μm)	Average	143	817	-	14.3	48.0	139	1150	2.12	114	-	[30]

**Table 2:** A comparison of heavy metal concentrations (mg·kg^−1^) in settled dust from different urban functional areas in Shijiazhuang.

Functional Area	No.	Cr	Mn	Fe	Co	Ni	Cu	Zn	Cd	Pb	V
		mg·kg^−^^1^									
North Railway Station	1	279.6	810.6	78,899	26.2	82.9	330.1	1485.8	6.12	470.0	114.9
Industrial area	16	143.2	555.2	41,630	14.7	43.0	101.2	560.0	3.58	173.2	63.2
Heavily trafficked area	8	142.8	571.1	44,948	15.1	40.5	103.9	471.2	2.70	228.6	67.0
Commercial area	4	126.8	539.4	43,548	14.5	43.3	112.2	476.4	2.13	180.0	66.7
Administration-education area	3	160.3	579.7	43,894	16.0	42.2	102.2	395.6	1.90	136.4	72.1
Other unclassified sites	11	125.2	547.1	41,228	13.9	37.8	97.9	442.1	2.02	128.2	62.7

## Abbreviations

The following abbreviations are used in this manuscript:

UCC	Upper Continental Crust
EF	Enrichment factor
IPI	Integrated pollution index
PI	Pollution index
